# Supplementary material for: Forecasting influenza epidemics by integrating internet search queries and traditional surveillance data with the support vector machine regression model in Liaoning, from 2011 to 2015
Source: PeerJ. 2018 Jun 25;6:e5134. doi: 10.7717/peerj.5134 (PMC6022725; doi:10.7717/peerj.5134)
Supplement: Supplemental Information 4 [file peerj-06-5134-s004.docx]

## the final included 17 search keywords and lag 1 month influenza casas are used for SVM regression model building. Raw data was included in Table S2.

Model training data was included in Table S2_1.

## **Parameter selection**

## **Parameter C**

**##c=0.0001**

z<-read.table("Table S2_1.csv",header=TRUE,sep=",")

x<-subset(z,select=-influenza.cases)

y<-subset(z,select= influenza.cases)

library(class)

library(e1071)

trainerror=0

testerror=0

for(i in 1:42)

{

xtrain1= x[-i,]

xtest1=x[i,]

ytrain1= y[-i,]

ytest1=y[i,]

model <- svm(xtrain1,ytrain1, cost= 0.0001,decision.value=TRUE,probability=TRUE)

svm.train.pred<-predict(model,xtrain1)

svm.test.pred <-predict(model, xtest1)

M<-c(svm.train.pred)

N<- c(svm.test.pred)

trainerror<- trainerror+ sum((M-ytrain1)^2)/41

testerror<-testerror+sum((N-ytest1)^2)/1

}

list(trainerror/42, testerror/42)

**##c=0.001**

z<-read.table("Table S2_1.csv",header=TRUE,sep=",")

x<-subset(z,select=-influenza.cases)

y<-subset(z,select= influenza.cases)

library(class)

library(e1071)

trainerror=0

testerror=0

for(i in 1:42)

{

xtrain1= x[-i,]

xtest1=x[i,]

ytrain1= y[-i,]

ytest1=y[i,]

model <- svm(xtrain1,ytrain1, cost = 0.001,decision.value=TRUE,probability=TRUE)

svm.train.pred<-predict(model,xtrain1)

svm.test.pred <-predict(model, xtest1)

M<-c(svm.train.pred)

N<- c(svm.test.pred)

trainerror<- trainerror+ sum((M-ytrain1)^2)

testerror<-testerror+sum((N-ytest1)^2)

}

list(trainerror, testerror)

**##c=0.01**

z<-read.table("Table S2_1.csv",header=TRUE,sep=",")

x<-subset(z,select=-influenza.cases)

y<-subset(z,select= influenza.cases)

library(class)

library(e1071)

trainerror=0

testerror=0

for(i in 1:42)

{

xtrain1= x[-i,]

xtest1=x[i,]

ytrain1= y[-i,]

ytest1=y[i,]

model <- svm(xtrain1,ytrain1, cost = 0.01,decision.value=TRUE,probability=TRUE)

svm.train.pred<-predict(model,xtrain1)

svm.test.pred <-predict(model, xtest1)

M<-c(svm.train.pred)

N<- c(svm.test.pred)

trainerror<- trainerror+ sum((M-ytrain1)^2)/41

testerror<-testerror+sum((N-ytest1)^2)/1

}

list(trainerror/42, testerror/42)

**##c=0.1**

z<-read.table("Table S2_1.csv",header=TRUE,sep=",")

x<-subset(z,select=-influenza.cases)

y<-subset(z,select= influenza.cases)

library(class)

library(e1071)

trainerror=0

testerror=0

for(i in 1:42)

{

xtrain1= x[-i,]

xtest1=x[i,]

ytrain1= y[-i,]

ytest1=y[i,]

model <- svm(xtrain1,ytrain1, cost = 0.1,decision.value=TRUE,probability=TRUE)

svm.train.pred<-predict(model,xtrain1)

svm.test.pred <-predict(model, xtest1)

M<-c(svm.train.pred)

N<- c(svm.test.pred)

trainerror<- trainerror+ sum((M-ytrain1)^2)/41

testerror<-testerror+sum((N-ytest1)^2)/1

}

list(trainerror/42, testerror/42)

**##c=1**

z<-read.table("Table S2_1.csv",header=TRUE,sep=",")

x<-subset(z,select=-influenza.cases)

y<-subset(z,select= influenza.cases)

library(class)

library(e1071)

trainerror=0

testerror=0

for(i in 1:42)

{

xtrain1= x[-i,]

xtest1=x[i,]

ytrain1= y[-i,]

ytest1=y[i,]

model <- svm(xtrain1,ytrain1, cost = 1,decision.value=TRUE,probability=TRUE)

svm.train.pred<-predict(model,xtrain1)

svm.test.pred <-predict(model, xtest1)

M<-c(svm.train.pred)

N<- c(svm.test.pred)

trainerror<- trainerror+ sum((M-ytrain1)^2)/41

testerror<-testerror+sum((N-ytest1)^2)/1

}

list(trainerror/42, testerror/42)

**##c=2**

z<-read.table("Table S2_1.csv",header=TRUE,sep=",")

x<-subset(z,select=-influenza.cases)

y<-subset(z,select= influenza.cases)

library(class)

library(e1071)

trainerror=0

testerror=0

for(i in 1:42)

{

xtrain1= x[-i,]

xtest1=x[i,]

ytrain1= y[-i,]

ytest1=y[i,]

model <- svm(xtrain1,ytrain1, cost = 2,decision.value=TRUE,probability=TRUE)

svm.train.pred<-predict(model,xtrain1)

svm.test.pred <-predict(model, xtest1)

M<-c(svm.train.pred)

N<- c(svm.test.pred)

trainerror<- trainerror+ sum((M-ytrain1)^2)/41

testerror<-testerror+sum((N-ytest1)^2)/1

}

list(trainerror/42, testerror/42)

**##c=3**

z<-read.table("Table S2_1.csv",header=TRUE,sep=",")

x<-subset(z,select=-influenza.cases)

y<-subset(z,select= influenza.cases)

library(class)

library(e1071)

trainerror=0

testerror=0

for(i in 1:42)

{

xtrain1= x[-i,]

xtest1=x[i,]

ytrain1= y[-i,]

ytest1=y[i,]

model <- svm(xtrain1,ytrain1, cost =3,decision.value=TRUE,probability=TRUE)

svm.train.pred<-predict(model,xtrain1)

svm.test.pred <-predict(model, xtest1)

M<-c(svm.train.pred)

N<- c(svm.test.pred)

trainerror<- trainerror+ sum((M-ytrain1)^2)/41

testerror<-testerror+sum((N-ytest1)^2)/1

}

list(trainerror/42, testerror/42)

**##c=4**

z<-read.table("Table S2_1.csv",header=TRUE,sep=",")

x<-subset(z,select=-influenza.cases)

y<-subset(z,select= influenza.cases)

library(class)

library(e1071)

trainerror=0

testerror=0

for(i in 1:42)

{

xtrain1= x[-i,]

xtest1=x[i,]

ytrain1= y[-i,]

ytest1=y[i,]

model <- svm(xtrain1,ytrain1, cost= 4,decision.value=TRUE,probability=TRUE)

svm.train.pred<-predict(model,xtrain1)

svm.test.pred <-predict(model, xtest1)

M<-c(svm.train.pred)

N<- c(svm.test.pred)

trainerror<- trainerror+ sum((M-ytrain1)^2)/41

testerror<-testerror+sum((N-ytest1)^2)/1

}

list(trainerror/42, testerror/42)

**##c=5**

z<-read.table("Table S2_1.csv",header=TRUE,sep=",")

x<-subset(z,select=-influenza.cases)

y<-subset(z,select= influenza.cases)

library(class)

library(e1071)

trainerror=0

testerror=0

for(i in 1:42)

{

xtrain1= x[-i,]

xtest1=x[i,]

ytrain1= y[-i,]

ytest1=y[i,]

model <- svm(xtrain1,ytrain1,cost = 5,decision.value=TRUE,probability=TRUE)

svm.train.pred<-predict(model,xtrain1)

svm.test.pred <-predict(model, xtest1)

M<-c(svm.train.pred)

N<- c(svm.test.pred)

trainerror<- trainerror+ sum((M-ytrain1)^2)/41

testerror<-testerror+sum((N-ytest1)^2)/1

}

list(trainerror/42, testerror/42)

**##c=10**

z<-read.table("Table S2_1.csv",header=TRUE,sep=",")

x<-subset(z,select=-influenza.cases)

y<-subset(z,select= influenza.cases)

library(class)

library(e1071)

trainerror=0

testerror=0

for(i in 1:42)

{

xtrain1= x[-i,]

xtest1=x[i,]

ytrain1= y[-i,]

ytest1=y[i,]

model <- svm(xtrain1,ytrain1, cost= 10,decision.value=TRUE,probability=TRUE)

svm.train.pred<-predict(model,xtrain1)

svm.test.pred <-predict(model, xtest1)

M<-c(svm.train.pred)

N<- c(svm.test.pred)

trainerror<- trainerror+ sum((M-ytrain1)^2)/41

testerror<-testerror+sum((N-ytest1)^2)/1

}

list(trainerror/42, testerror/42)

**##c=100**

z<-read.table("Table S2_1.csv",header=TRUE,sep=",")

x<-subset(z,select=-influenza.cases)

y<-subset(z,select= influenza.cases)

library(class)

library(e1071)

trainerror=0

testerror=0

for(i in 1:42)

{

xtrain1= x[-i,]

xtest1=x[i,]

ytrain1= y[-i,]

ytest1=y[i,]

model <- svm(xtrain1,ytrain1, cost= 100,decision.value=TRUE,probability=TRUE)

svm.train.pred<-predict(model,xtrain1)

svm.test.pred <-predict(model, xtest1)

M<-c(svm.train.pred)

N<- c(svm.test.pred)

trainerror<- trainerror+ sum((M-ytrain1)^2)/41

testerror<-testerror+sum((N-ytest1)^2)/1

}

list(trainerror/42, testerror/42)

## **Parameter γ**

**##gamma=0.0001**

z<-read.table("Table S2_1.csv",header=TRUE,sep=",")

x<-subset(z,select=-influenza.cases)

y<-subset(z,select= influenza.cases)

library(class)

library(e1071)

trainerror=0

testerror=0

for(i in 1:42)

{

xtrain1= x[-i,]

xtest1=x[i,]

ytrain1= y[-i,]

ytest1=y[i,]

model <- svm(xtrain1,ytrain1, gamma = 0.0001,decision.value=TRUE,probability=TRUE)

svm.train.pred<-predict(model,xtrain1)

svm.test.pred <-predict(model, xtest1)

M<-c(svm.train.pred)

N<- c(svm.test.pred)

trainerror<- trainerror+ sum((M-ytrain1)^2)/41

testerror<-testerror+sum((N-ytest1)^2)/1

}

list(trainerror/42, testerror/42)

**##gamma=0.001**

z<-read.table("Table S2_1.csv",header=TRUE,sep=",")

x<-subset(z,select=-influenza.cases)

y<-subset(z,select= influenza.cases)

library(class)

library(e1071)

trainerror=0

testerror=0

for(i in 1:42)

{

xtrain1= x[-i,]

xtest1=x[i,]

ytrain1= y[-i,]

ytest1=y[i,]

model <- svm(xtrain1,ytrain1, gamma = 0.001,decision.value=TRUE,probability=TRUE)

svm.train.pred<-predict(model,xtrain1)

svm.test.pred <-predict(model, xtest1)

M<-c(svm.train.pred)

N<- c(svm.test.pred)

trainerror<- trainerror+ sum((M-ytrain1)^2)/41

testerror<-testerror+sum((N-ytest1)^2)/1

}

list(trainerror/42, testerror/42)

**##gamma=0.005**

z<-read.table("Table S2_1.csv",header=TRUE,sep=",")

x<-subset(z,select=-influenza.cases)

y<-subset(z,select= influenza.cases)

library(class)

library(e1071)

trainerror=0

testerror=0

for(i in 1:42)

{

xtrain1= x[-i,]

xtest1=x[i,]

ytrain1= y[-i,]

ytest1=y[i,]

model <- svm(xtrain1,ytrain1, gamma = 0.005,decision.value=TRUE,probability=TRUE)

svm.train.pred<-predict(model,xtrain1)

svm.test.pred <-predict(model, xtest1)

M<-c(svm.train.pred)

N<- c(svm.test.pred)

trainerror<- trainerror+ sum((M-ytrain1)^2)/41

testerror<-testerror+sum((N-ytest1)^2)/1

}

list(trainerror/42, testerror/42)

**##gamma=0.01**

z<-read.table("Table S2_1.csv",header=TRUE,sep=",")

x<-subset(z,select=-influenza.cases)

y<-subset(z,select= influenza.cases)

library(class)

library(e1071)

trainerror=0

testerror=0

for(i in 1:42)

{

xtrain1= x[-i,]

xtest1=x[i,]

ytrain1= y[-i,]

ytest1=y[i,]

model <- svm(xtrain1,ytrain1, gamma = 0.01,decision.value=TRUE,probability=TRUE)

svm.train.pred<-predict(model,xtrain1)

svm.test.pred <-predict(model, xtest1)

M<-c(svm.train.pred)

N<- c(svm.test.pred)

trainerror<- trainerror+ sum((M-ytrain1)^2)/41

testerror<-testerror+sum((N-ytest1)^2)/1

}

list(trainerror/42, testerror/42)

**##gamma=0.02**

z<-read.table("Table S2_1.csv",header=TRUE,sep=",")

x<-subset(z,select=-influenza.cases)

y<-subset(z,select= influenza.cases)

library(class)

library(e1071)

trainerror=0

testerror=0

for(i in 1:42)

{

xtrain1= x[-i,]

xtest1=x[i,]

ytrain1= y[-i,]

ytest1=y[i,]

model <- svm(xtrain1,ytrain1, gamma = 0.02,decision.value=TRUE,probability=TRUE)

svm.train.pred<-predict(model,xtrain1)

svm.test.pred <-predict(model, xtest1)

M<-c(svm.train.pred)

N<- c(svm.test.pred)

trainerror<- trainerror+ sum((M-ytrain1)^2)/41

testerror<-testerror+sum((N-ytest1)^2)/1

}

list(trainerror/42, testerror/42)

**##gamma=0.03**

z<-read.table("Table S2_1.csv",header=TRUE,sep=",")

x<-subset(z,select=-influenza.cases)

y<-subset(z,select= influenza.cases)

library(class)

library(e1071)

trainerror=0

testerror=0

for(i in 1:42)

{

xtrain1= x[-i,]

xtest1=x[i,]

ytrain1= y[-i,]

ytest1=y[i,]

model <- svm(xtrain1,ytrain1, gamma = 0.03,decision.value=TRUE,probability=TRUE)

svm.train.pred<-predict(model,xtrain1)

svm.test.pred <-predict(model, xtest1)

M<-c(svm.train.pred)

N<- c(svm.test.pred)

trainerror<- trainerror+ sum((M-ytrain1)^2)/41

testerror<-testerror+sum((N-ytest1)^2)/1

}

list(trainerror/42, testerror/42)

**##gamma=0.04**

z<-read.table("Table S2_1.csv",header=TRUE,sep=",")

x<-subset(z,select=-influenza.cases)

y<-subset(z,select= influenza.cases)

library(class)

library(e1071)

trainerror=0

testerror=0

for(i in 1:42)

{

xtrain1= x[-i,]

xtest1=x[i,]

ytrain1= y[-i,]

ytest1=y[i,]

model <- svm(xtrain1,ytrain1, gamma = 0.04,decision.value=TRUE,probability=TRUE)

svm.train.pred<-predict(model,xtrain1)

svm.test.pred <-predict(model, xtest1)

M<-c(svm.train.pred)

N<- c(svm.test.pred)

trainerror<- trainerror+ sum((M-ytrain1)^2)/41

testerror<-testerror+sum((N-ytest1)^2)/1

}

list(trainerror/42, testerror/42)

**##gamma=0.05**

z<-read.table("Table S2_1.csv",header=TRUE,sep=",")

x<-subset(z,select=-influenza.cases)

y<-subset(z,select= influenza.cases)

library(class)

library(e1071)

trainerror=0

testerror=0

for(i in 1:42)

{

xtrain1= x[-i,]

xtest1=x[i,]

ytrain1= y[-i,]

ytest1=y[i,]

model <- svm(xtrain1,ytrain1, gamma = 0.05,decision.value=TRUE,probability=TRUE)

svm.train.pred<-predict(model,xtrain1)

svm.test.pred <-predict(model, xtest1)

M<-c(svm.train.pred)

N<- c(svm.test.pred)

trainerror<- trainerror+ sum((M-ytrain1)^2)/41

testerror<-testerror+sum((N-ytest1)^2)/1

}

list(trainerror/42, testerror/42)

**##gamma=0.1**

z<-read.table("Table S2_1.csv",header=TRUE,sep=",")

x<-subset(z,select=-influenza.cases)

y<-subset(z,select= influenza.cases)

library(class)

library(e1071)

trainerror=0

testerror=0

for(i in 1:42)

{

xtrain1= x[-i,]

xtest1=x[i,]

ytrain1= y[-i,]

ytest1=y[i,]

model <- svm(xtrain1,ytrain1, gamma = 0.1,decision.value=TRUE,probability=TRUE)

svm.train.pred<-predict(model,xtrain1)

svm.test.pred <-predict(model, xtest1)

M<-c(svm.train.pred)

N<- c(svm.test.pred)

trainerror<- trainerror+ sum((M-ytrain1)^2)/41

testerror<-testerror+sum((N-ytest1)^2)/1

}

list(trainerror/42, testerror/42)

**##gamma=1**

z<-read.table("Table S2_1.csv",header=TRUE,sep=",")

x<-subset(z,select=-influenza.cases)

y<-subset(z,select= influenza.cases)

library(class)

library(e1071)

trainerror=0

testerror=0

for(i in 1:42)

{

xtrain1= x[-i,]

xtest1=x[i,]

ytrain1= y[-i,]

ytest1=y[i,]

model <- svm(xtrain1,ytrain1, gamma = 1,decision.value=TRUE,probability=TRUE)

svm.train.pred<-predict(model,xtrain1)

svm.test.pred <-predict(model, xtest1)

M<-c(svm.train.pred)

N<- c(svm.test.pred)

trainerror<- trainerror+ sum((M-ytrain1)^2)/41

testerror<-testerror+sum((N-ytest1)^2)/1

}

list(trainerror/42, testerror/42)

## **Parameter ε**

**##epsilon=0.0001**

z<-read.table("Table S2_1.csv",header=TRUE,sep=",")

x<-subset(z,select=-influenza.cases)

y<-subset(z,select= influenza.cases)

library(class)

library(e1071)

trainerror=0

testerror=0

for(i in 1:42)

{

xtrain1= x[-i,]

xtest1=x[i,]

ytrain1= y[-i,]

ytest1=y[i,]

model <- svm(xtrain1,ytrain1, epsilon=0.0001,decision.value=TRUE,probability=TRUE)

svm.train.pred<-predict(model,xtrain1)

svm.test.pred <-predict(model, xtest1)

M<-c(svm.train.pred)

N<- c(svm.test.pred)

trainerror<- trainerror+ sum((M-ytrain1)^2)/41

testerror<-testerror+sum((N-ytest1)^2)/1

}

list(trainerror/42, testerror/42)

**##epsilon=0.001**

z<-read.table("Table S2_1.csv",header=TRUE,sep=",")

x<-subset(z,select=-influenza.cases)

y<-subset(z,select= influenza.cases)

library(class)

library(e1071)

trainerror=0

testerror=0

for(i in 1:42)

{

xtrain1= x[-i,]

xtest1=x[i,]

ytrain1= y[-i,]

ytest1=y[i,]

model <- svm(xtrain1,ytrain1, epsilon=0.001,decision.value=TRUE,probability=TRUE)

svm.train.pred<-predict(model,xtrain1)

svm.test.pred <-predict(model, xtest1)

M<-c(svm.train.pred)

N<- c(svm.test.pred)

trainerror<- trainerror+ sum((M-ytrain1)^2)/41

testerror<-testerror+sum((N-ytest1)^2)/1

}

list(trainerror/42, testerror/42)

**##epsilon=0.01**

z<-read.table("Table S2_1.csv",header=TRUE,sep=",")

x<-subset(z,select=-influenza.cases)

y<-subset(z,select= influenza.cases)

library(class)

library(e1071)

trainerror=0

testerror=0

for(i in 1:42)

{

xtrain1= x[-i,]

xtest1=x[i,]

ytrain1= y[-i,]

ytest1=y[i,]

model <- svm(xtrain1,ytrain1, epsilon=0.01,decision.value=TRUE,probability=TRUE)

svm.train.pred<-predict(model,xtrain1)

svm.test.pred <-predict(model, xtest1)

M<-c(svm.train.pred)

N<- c(svm.test.pred)

trainerror<- trainerror+ sum((M-ytrain1)^2)/41

testerror<-testerror+sum((N-ytest1)^2)/1

}

list(trainerror/42, testerror/42)

**##epsilon=0.05**

z<-read.table("Table S2_1.csv",header=TRUE,sep=",")

x<-subset(z,select=-influenza.cases)

y<-subset(z,select= influenza.cases)

library(class)

library(e1071)

trainerror=0

testerror=0

for(i in 1:42)

{

xtrain1= x[-i,]

xtest1=x[i,]

ytrain1= y[-i,]

ytest1=y[i,]

model <- svm(xtrain1,ytrain1, epsilon=0.05,decision.value=TRUE,probability=TRUE)

svm.train.pred<-predict(model,xtrain1)

svm.test.pred <-predict(model, xtest1)

M<-c(svm.train.pred)

N<- c(svm.test.pred)

trainerror<- trainerror+ sum((M-ytrain1)^2)/41

testerror<-testerror+sum((N-ytest1)^2)/1

}

list(trainerror/42, testerror/42)

**##epsilon=0.08**

z<-read.table("Table S2_1.csv",header=TRUE,sep=",")

x<-subset(z,select=-influenza.cases)

y<-subset(z,select= influenza.cases)

library(class)

library(e1071)

trainerror=0

testerror=0

for(i in 1:42)

{

xtrain1= x[-i,]

xtest1=x[i,]

ytrain1= y[-i,]

ytest1=y[i,]

model <- svm(xtrain1,ytrain1, epsilon=0.08,decision.value=TRUE,probability=TRUE)

svm.train.pred<-predict(model,xtrain1)

svm.test.pred <-predict(model, xtest1)

M<-c(svm.train.pred)

N<- c(svm.test.pred)

trainerror<- trainerror+ sum((M-ytrain1)^2)/41

testerror<-testerror+sum((N-ytest1)^2)/1

}

list(trainerror/42, testerror/42)

**##epsilon=0.09**

z<-read.table("Table S2_1.csv",header=TRUE,sep=",")

x<-subset(z,select=-influenza.cases)

y<-subset(z,select= influenza.cases)

library(class)

library(e1071)

trainerror=0

testerror=0

for(i in 1:42)

{

xtrain1= x[-i,]

xtest1=x[i,]

ytrain1= y[-i,]

ytest1=y[i,]

model <- svm(xtrain1,ytrain1, epsilon=0.09,decision.value=TRUE,probability=TRUE)

svm.train.pred<-predict(model,xtrain1)

svm.test.pred <-predict(model, xtest1)

M<-c(svm.train.pred)

N<- c(svm.test.pred)

trainerror<- trainerror+ sum((M-ytrain1)^2)/41

testerror<-testerror+sum((N-ytest1)^2)/1

}

list(trainerror/42, testerror/42)

**##epsilon=0.1**

z<-read.table("Table S2_1.csv",header=TRUE,sep=",")

x<-subset(z,select=-influenza.cases)

y<-subset(z,select= influenza.cases)

library(class)

library(e1071)

trainerror=0

testerror=0

for(i in 1:42)

{

xtrain1= x[-i,]

xtest1=x[i,]

ytrain1= y[-i,]

ytest1=y[i,]

model <- svm(xtrain1,ytrain1, epsilon=0.1,decision.value=TRUE,probability=TRUE)

svm.train.pred<-predict(model,xtrain1)

svm.test.pred <-predict(model, xtest1)

M<-c(svm.train.pred)

N<- c(svm.test.pred)

trainerror<- trainerror+ sum((M-ytrain1)^2)/41

testerror<-testerror+sum((N-ytest1)^2)/1

}

list(trainerror/42, testerror/42)

**##epsilon=0.2**

z<-read.table("Table S2_1.csv",header=TRUE,sep=",")

x<-subset(z,select=-influenza.cases)

y<-subset(z,select= influenza.cases)

library(class)

library(e1071)

trainerror=0

testerror=0

for(i in 1:42)

{

xtrain1= x[-i,]

xtest1=x[i,]

ytrain1= y[-i,]

ytest1=y[i,]

model <- svm(xtrain1,ytrain1, epsilon=0.2,decision.value=TRUE,probability=TRUE)

svm.train.pred<-predict(model,xtrain1)

svm.test.pred <-predict(model, xtest1)

M<-c(svm.train.pred)

N<- c(svm.test.pred)

trainerror<- trainerror+ sum((M-ytrain1)^2)/41

testerror<-testerror+sum((N-ytest1)^2)/1

}

list(trainerror/42, testerror/42)

**##epsilon=0.3**

z<-read.table("Table S2_1.csv",header=TRUE,sep=",")

x<-subset(z,select=-influenza.cases)

y<-subset(z,select= influenza.cases)

library(class)

library(e1071)

trainerror=0

testerror=0

for(i in 1:42)

{

xtrain1= x[-i,]

xtest1=x[i,]

ytrain1= y[-i,]

ytest1=y[i,]

model <- svm(xtrain1,ytrain1, epsilon=0.3,decision.value=TRUE,probability=TRUE)

svm.train.pred<-predict(model,xtrain1)

svm.test.pred <-predict(model, xtest1)

M<-c(svm.train.pred)

N<- c(svm.test.pred)

trainerror<- trainerror+ sum((M-ytrain1)^2)/41

testerror<-testerror+sum((N-ytest1)^2)/1

}

list(trainerror/42, testerror/42)

**##epsilon=0.4**

z<-read.table("Table S2_1.csv",header=TRUE,sep=",")

x<-subset(z,select=-influenza.cases)

y<-subset(z,select= influenza.cases)

library(class)

library(e1071)

trainerror=0

testerror=0

for(i in 1:42)

{

xtrain1= x[-i,]

xtest1=x[i,]

ytrain1= y[-i,]

ytest1=y[i,]

model <- svm(xtrain1,ytrain1, epsilon=0.4,decision.value=TRUE,probability=TRUE)

svm.train.pred<-predict(model,xtrain1)

svm.test.pred <-predict(model, xtest1)

M<-c(svm.train.pred)

N<- c(svm.test.pred)

trainerror<- trainerror+ sum((M-ytrain1)^2)/41

testerror<-testerror+sum((N-ytest1)^2)/1

}

list(trainerror/42, testerror/42)

**##epsilon=0.5**

z<-read.table("Table S2_1.csv",header=TRUE,sep=",")

x<-subset(z,select=-influenza.cases)

y<-subset(z,select= influenza.cases)

library(class)

library(e1071)

trainerror=0

testerror=0

for(i in 1:42)

{

xtrain1= x[-i,]

xtest1=x[i,]

ytrain1= y[-i,]

ytest1=y[i,]

model <- svm(xtrain1,ytrain1, epsilon=0.5,decision.value=TRUE,probability=TRUE)

svm.train.pred<-predict(model,xtrain1)

svm.test.pred <-predict(model, xtest1)

M<-c(svm.train.pred)

N<- c(svm.test.pred)

trainerror<- trainerror+ sum((M-ytrain1)^2)/41

testerror<-testerror+sum((N-ytest1)^2)/1

}

list(trainerror/42, testerror/42)

**##epsilon=1**

z<-read.table("Table S2_1.csv",header=TRUE,sep=",")

x<-subset(z,select=-influenza.cases)

y<-subset(z,select= influenza.cases)

library(class)

library(e1071)

trainerror=0

testerror=0

for(i in 1:42)

{

xtrain1= x[-i,]

xtest1=x[i,]

ytrain1= y[-i,]

ytest1=y[i,]

model <- svm(xtrain1,ytrain1, epsilon=1,decision.value=TRUE,probability=TRUE)

svm.train.pred<-predict(model,xtrain1)

svm.test.pred <-predict(model, xtest1)

M<-c(svm.train.pred)

N<- c(svm.test.pred)

trainerror<- trainerror+ sum((M-ytrain1)^2)/41

testerror<-testerror+sum((N-ytest1)^2)/1

}

list(trainerror/42, testerror/42)

## **Comparison and prediction of SVM regression models from different data source.**

**##** lag one month's influenza cases data source

z<-read.table("Table S2.csv",header=TRUE,sep=",")

x<-subset(z,select= lag.one.month.influenza.cases)

y<-subset(z,select= influenza.cases)

xtrain<-x[4:45,]

ytrain<-y[4:45,]

xtest<-x[46:60,]

ytest<-y[46:60,]

model<-svm(xtrain,ytrain, cost=2, gamma=0.005, epsilon=0.001,decision.value=TRUE,probability=TRUE)

predl<-predict(model,xtest)

summary(model)

predl

## the Baidu search data source

z<-read.table("Table S2.csv",header=TRUE,sep=",")

x<-subset(z,select=- lag.one.month.influenza.cases)

x<-subset(x,select=- influenza.cases)

y<-subset(z,select= influenza.cases)

xtrain<-x[4:45,]

ytrain<-y[4:45,]

xtest<-x[46:60,]

ytest<-y[46:60,]

model<-svm(xtrain,ytrain, cost=2, gamma=0.005, epsilon=0.001,decision.value=TRUE,probability=TRUE)

predw<-predict(model,xtest)

summary(model)

predw

## ensemble data integrating past influenza cases data and Baidu search data

z<-read.table("Table S2.csv",header=TRUE,sep=",")

x<-subset(z,select=- influenza.cases)

y<-subset(z,select= influenza.cases)

xtrain<-x[4:45,]

ytrain<-y[4:45,]

xtest<-x[46:60,]

ytest<-y[46:60,]

model<-svm(xtrain,ytrain, cost=2, gamma=0.005, epsilon=0.001,decision.value=TRUE,probability=TRUE)

predR<-predict(model,xtest)

summary(model)

predR

list(predl,predw,predR)
